# Analysis of the relationship between shorter sleep duration and wrist fractures: based on NHANES

**DOI:** 10.1186/s12891-024-07497-9

**Published:** 2024-05-14

**Authors:** Lang Wu, Shaoyu Han, Bingjun Cui, Chuangong Wang, Zhenqing Zhang, Zhixiang Chen

**Affiliations:** 1 Trauma Center, Huai’an Hospital Affiliated to Yangzhou University (The Fifth People’s Hospital of Huai’an), Huai’an, Jiangsu Province 223001 China; 2 Department of Orthopedic Surgery, Northern Jiangsu People’s Hospital Affiliated to Yangzhou University, Yangzhou, Jiangsu Province 225001 China

**Keywords:** Wrist fracture, Shorter sleep duration, NHANES data

## Abstract

**Background:**

Wrist fracture is one of the common limb fractures. Its incidence rate increases with age and osteoporosis. Nowadays, Sleep health is increasingly valued, but the relationship between wrist fractures and sleep time is not yet clear.

**Methods:**

Data in this study were collected and screened from the NHANES from 2005 to 2010 and 2013 to 2014. The variables were extracted from interviews and compared between the wrist fractures and the sleep duration. The data was analyzed by weighted multivariate logistic regression.

**Results:**

After excluding individuals who were not eligible and had invalid data, we finally identified 1835 participants for inclusion in this study. We found a negative association between the sleep duration and the fractured of the wrist (OR = 1.027,95% CI (1.027, 1.028), *P* < 0.00001).

**Conclusion:**

This study demons that the association between the sleep duration and the fractures of the wrist is significant. Our findings provide a better understanding of the relationship between sleep duration and wrist fractures. This study may help us reducing the incidence of wrist fractures in the population based on healthy sleep management in the future, and improve the quality of life of middle-aged and elderly patients. Provide evidence for clinical patients to manage healthy sleep.

## Introduction

Wrist fracture is one of the common limb fractures. Its incidence rate increases with age, and its incidence rate increases year by year with the increase of the elderly population [1]. Among the wrist fractures, the distal radius fractures are more common, accounting for 92-95% [2]. The distal radius fractures often lead to acute pain and functional impairment. If left untreated or treated improperly, it can seriously affect the function of the patient’s wrist and even have a significant impact on their quality of life [3, 4]. In the United States, the incidence of wrist fractures is three times higher than that of hip fractures. Therefore, more and more people are paying attention to wrist fractures [5, 6].

Fractures of the distal radius are common in elderly women, especially in the presence of osteoporosis, usually occurring after falling and extending the arm to support the ground. Low-energy injuries such as slipping and missing steps are the main causes of injury.

Current research has found that the duration of sleep is associated with osteoporosis [7, 8]. A growing amount of evidence shows that the adequate sleep is very important for health. Kuriyama.

’s study showed that short sleep duration(≤ 5 hours) caused malregulation of the sympathetic nervous system. Based on the malregulation, it will cause a loss of cortical bone thickness [8]. Meanwhile, Swanson also found that the ciecadian system and sleep was important for bone health based on the disruption of bone turnover markers [9]. The National Sleep Foundation suggests sleeping more than seven hours per day is recommended to mitigate adverse impacts of short sleep duration on bone health [10].

From our clinical data, we found that wrist fractures often happened in the people who get short sleep duration. Reviewing recent studies, there is currently no clinical research on the relationship between sleep duration and wrist fractures. This study aims to investigate the association between sleep duration and wrist fractures through a retrospective analysis of data from the United States National Health and Nutrition Examination Survey (NHANES) from 2005 to 2010 and 2013–2014.

This study analyzed the public data from the Examination Survey to explore the relationship between reduced sleep time and the incidence of wrist fractures in people over 50 years old, providing new ideas for the prevention of wrist fractures.

## Methods

### Study design and population

**Fig. 1:**
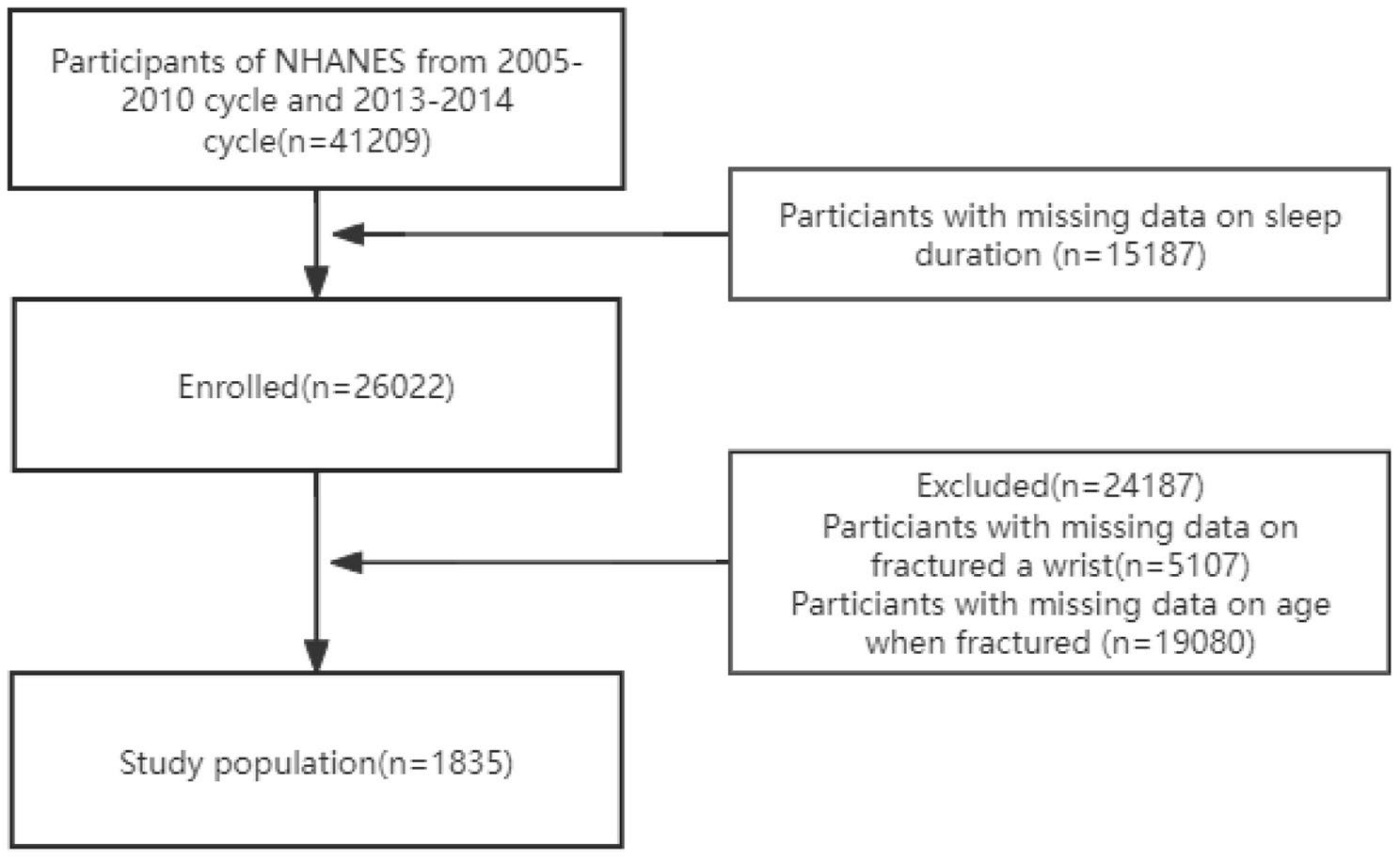
Screening procedure flowchart

The United States National Health and Nutrition Examination Survey is a survey conducted by the Centers for Disease Prevention and Control in the United States. The CDC organizes a large-scale health survey project every two years, and its data is publicly available worldwide for free use. We searched and downloaded the health data of the 2005–2010 cycle and 2013–2014 cycle on its website. The recruitment details, recruitment procedures, population characteristics, and research design of the surveyed population were provided by the CDC. All participants completed a family interview, and the observers came from a unified team and implemented a unified interview standard, which significantly reduced the differences caused by observers. The data showed that a total of 41,209 patients were recruited to participate in the questionnaire, physical examination and evaluation. After excluding people with sleep time, wrist fractures, age less than 50 years old with wrist fractures, and missing information on age of wrist fractures, a total of 1835 people were included in this study, of which 1480 people had sleep time greater than 5 h, while 355 people had sleep time less than or equal to 5 hours (Fig. 1). The relevant information of the above-mentioned people was used for further research. The datasets generated and analyzed during the current study are available in the organizational website (www.cdc.gov/nchs/nhanes), or required from the corresponding author.

### Study variables

The data were collected through standardized questionnaires and medical data from medical centers. Combining the characteristics of previous epidemiological studies of wrist fractures and the variables provided by the NHANES data, we collected the following data from the subjects as variables: gender, whether or not they had a wrist fracture, race, education level, marital status, and average smoking volume in the past 30 days. We kept the data that the key variables such as the length of sleep, the presence or absence of wrist fractures, and the age at which wrist fractures occurred were not missed. The variables which have complete key variables but have missing data in others was adjusted continuous variables to categorical variables for analysis.

### Statistical analysis

After data screening, continuous variables were expressed as mean standard, enumeration data were expressed as percentage (%), t-test was used for continuous variables, and chi-square test was used for categorical variables. Continuous variables are expressed as mean (95%CI), while categorical variables are expressed as count(percentage). All data were weighted to produce estimates for the US population, and designed layering and clustering were used in the analysis. In addition, variables such as age at the time of wrist fracture, gender, race, education, marital status, smoking consumption. The data analysis in this study took into account sampling weights based on the analytical guideline edited by NCHS, and was conducted using package R version 3.43 (http://www.R-project.org) and EmpowerStats software (http://www.empowerstats.com). We defined that *p*<0.05 was regarded as significant.

## Results

### Clinical characteristics

The clinical characteristics of this study population are shown in Table 1. A total of 1835 people slept for more than 5 h, 1480 people slept for more than 5 h, and 355 people slept for less than or equal to 5 h. There were no significant differences in age, gender, and average smoking volume over the past 30 days between the two groups. However, there were significant differences between the two groups in terms of race, educational background, and marital status (*P* < 0.05).

**Table 1:** General characteristics of participants

	Sleep Duration			
	> 5	<=5	Standardize diff.	*P* value
AGE	50.1 ± 17.1	49.0 ± 16.2	0.069 (0.068, 0.069)	0.2811
GENDER				0.6407
Male	54.1	55.5	0.029 (0.029, 0.030)	
Female	45.9	44.5	0.029 (0.029, 0.030)	
RACE				< 0.0001
Mexican American	4.3	4.9	0.028 (0.027, 0.029)	
Other Hispanic	2.2	3	0.049 (0.049, 0.050)	
Non-Hispanic White	84.9	74.2	0.267 (0.266, 0.268)	
Non-Hispanic Black	5	10.6	0.210 (0.209, 0.211)	
Other Race	3.6	7.3	0.164 (0.163, 0.164)	
EDUCATION				< 0.0001
Less Than 9th Grade	4.6	6.5	0.081 (0.081, 0.082)	
9-11th Grade	11.6	15.3	0.109 (0.108, 0.110)	
High School Grad	21.3	31.9	0.240 (0.240, 0.241)	
Some College or AA degree	34.1	35.3	0.026 (0.026, 0.027)	
College Graduate or above	28.4	11	0.448 (0.447, 0.448)	
MARITAL STATUS				< 0.0001
Married	57.9	50.8	0.143 (0.143, 0.144)	
Widowed	6.8	11.2	0.154 (0.154, 0.155)	
Divorced	11.5	11.2	0.009 (0.009, 0.010)	
Separated	1.8	5.6	0.206 (0.206, 0.207)	
Never married	16	12.1	0.114 (0.113, 0.115)	
Living with partner	6	9.1	0.117 (0.116, 0.117)	
Average alcoholic drinks/day - past 12 mos	3.0 ± 3.1	7.6 ± 65.4	0.100 (0.100, 0.101)	0.0307
Average cigarettes/day during past 30 days	16.1 ± 28.7	19.9 ± 15.3	0.164 (0.164, 0.165)	0.1491
Under/over 50 when fractured wrist				0.4992
Under	84.6	86.1	0.043 (0.042, 0.043)	
Over	15.4	13.9	0.043 (0.042, 0.043)	

### The association between wrist fractures and population characteristics

The characteristics of the enrolled individuals are presented by using linear regression in Table 2. We found that the correlation was non-significant(*P* = 0.381). Statistics have found that the length of sleep time is negatively correlated with the occurrence of wrist fractures. When sleep time is grouped, the OR values are:1.0, 1.043 (1.043, 1.044) < 0.00001 (P for trend 0.022), after adjusting for basic demographic variables (age, gender, race), the OR values were: 1.0, 1.059 (1.058, 1.059) < 0.00001 (P for trend 0.001), after further adjustment, the OR values were: 1.0, 1.013 (1.012, 1.015) < 0.00001 (P for trend < 0.001) (Table 3).

**Table 2:** Mean the variables: adjusted the age, race, and gender

AGE quartile	Variable	Correlation	β (95%CI)	*P*
Q1	RACE	0.0389	0.0477(-0.0547,0.1324)	0.416073
Q1	GENDER	-0.1948	0.0469(-0.2867,-0.103)	0.000039
Q2	RACE	0.0145	0.0474(-0.0784,0.1074)	0.759752
Q2	GENDER	-0.037	0.0474(-0.1298,0.0559)	0.435526
Q3	RACE	0.069	0.0461(-0.0214,0.1594)	0.135287
Q3	GENDER	0.0501	0.0462(-0.0404,0.1406)	0.278655
Q4	RACE	0.0177	0.046(-0.0725,0.1079)	0.700248
Q4	GENDER	-0.0167	0.046(-0.1069,0.0735)	0.716713
GENDER				
Male	AGE. Q4: AGE quartile	0.0329	0.0322(-0.0301,0.096)	0.306066
Male	RACE	0.0202	0.0322(-0.0429,0.0832)	0.531259
Female	AGE. Q4: AGE quartile	0.0435	0.034(-0.0232,0.1102)	0.201598
Female	RACE	0.0439	0.034(-0.0228,0.1106)	0.197699
RACE				
Mexican American	AGE. Q4: AGE quartile	0.7001	0.0494(0.6033,0.7969)	< 0.000001
Mexican American	GENDER	0.3061	0.0659(0.1771,0.4352)	0.000006
Other Hispanic	AGE. Q4: AGE quartile	0.5999	0.0792(0.4447,0.7552)	< 0.000001
Other Hispanic	GENDER	0.3056	0.0943(0.1208,0.4904)	0.001607
Non-Hispanic White	AGE. Q4: AGE quartile	0.0351	0.0287(-0.0212,0.0914)	0.221591
Non-Hispanic White	GENDER	-0.0017	0.0287(-0.058,0.0546)	0.952106
Non-Hispanic Black	AGE. Q4: AGE quartile	0.086	0.066(-0.0433,0.2154)	0.193541
Non-Hispanic Black	GENDER	0.0054	0.0662(-0.1244,0.1352)	0.935346
Other Race	AGE. Q4: AGE quartile	0.055	0.1193(-0.1789,0.2889)	0.646341
Other Race	GENDER	0.0799	0.1191(-0.1536,0.3134)	0.504599
Age when fractured wrist	How much sleep do you get (hours)? continuous categorical	0.0205	0.0234(-0.0253,0.0663)	0.380735

**Table 3:** Association between sleep duration and wrist fracture among U.S. adults

	Crude model	Model1	Model2
How much sleep do you get (hours)? continuous	1.007 (1.007, 1.007) < 0.00001	0.981 (0.981, 0.981) < 0.00001	1.027 (1.027, 1.028) < 0.00001
Categorical			
> 5	1.0	1.0	1.0
<=5	1.043 (1.043, 1.044) < 0.00001	1.059 (1.058, 1.059) < 0.00001	1.013 (1.012, 1.015) < 0.00001
P for trend	0.022	0.001	< 0.001

Model 1: None Adjusted

Model 2: adjusted for Gender; Race; Age when fractured wrist 1st time

Model 3: adjusted for: Gender; Race; Education; Marital Status; Average alcoholic drinks/day - past 12 mos; Age when fractured wrist 1st time; Average cigarettes/day during past 30 days

**Table 4:** Association between sleep duration and wrist fracture among U.S. adults adjusted by basic characters

	Crude model		*P* for trend	Model1			Model2		
	How much sleep do you get (hours)? continuous categorical			How much sleep do you get (hours)? continuous categorical			How much sleep do you get (hours)? continuous categorical		
	> 5	<=5		> 5	<=5		> 5	<=5	
Gender	1.0	1.047 (1.046, 1.047) < 0.00001	0.340	1.0	1.059 (1.058, 1.059) < 0.00001	0.201	1.0	1.013 (1.012, 1.015) < 0.00001	0.883
Male	1.0	0.987 (0.986, 0.987) < 0.00001	0.837	1.0	1.004 (1.004, 1.005) < 0.00001	0.972	1.0	1.010 (1.008, 1.011) < 0.00001	0.951
Female	1.0	1.107 (1.106, 1.108) < 0.00001	0.137	1.0	1.099 (1.098, 1.100) < 0.00001	0.124	1.0	1.015 (1.013, 1.017) < 0.00001	0.835
Age 2	1.0	1.087 (1.087, 1.088) < 0.00001	0.051	1.0	1.068 (1.067, 1.069) < 0.00001	0.109	1.0	1.022 (1.021, 1.024) < 0.00001	0.919
Q1	1.0	1.092 (1.090, 1.093) < 0.00001	0.463	1.0	1.055 (1.054, 1.057) < 0.00001	0.637	1.0	1.021 (1.019, 1.024) < 0.00001	0.896
Q2	1.0	1.188 (1.187, 1.189) < 0.00001	0.058	1.0	1.155 (1.153, 1.156) < 0.00001	0.118	1.0	1.219 (1.217, 1.221) < 0.00001	0.308
Q3	1.0	1.005 (1.005, 1.006) < 0.00001	0.945	1.0	0.937 (0.937, 0.938) < 0.00001	0.392	1.0	0.846 (0.843, 0.849) < 0.00001	0.495
Q4	1.0	1.083 (1.082, 1.084) < 0.00001	0.210	1.0	1.067 (1.066, 1.068) < 0.00001	0.288	1.0	0.000 (0.000, Inf) 0.99426	NaN
Race 3	1.0	1.031 (1.031, 1.032) < 0.00001	0.524	1.0	1.059 (1.058, 1.059) < 0.00001	0.201	1.0	0.906 (0.905, 0.907) < 0.00001	0.883
Mexican American	1.0	1.212 (1.209, 1.215) < 0.00001	0.172	1.0	0.946 (0.944, 0.948) < 0.00001	0.944	1.0	0.735 (0.727, 0.743) < 0.00001	0.680
Other Hispanic	1.0	1.092 (1.088, 1.095) < 0.00001	0.625	1.0	0.978 (0.975, 0.981) < 0.00001	0.825	1.0	9.492 (9.287, 9.701) < 0.00001	0.105
Non-Hispanic White	1.0	1.047 (1.047, 1.048) < 0.00001	0.461	1.0	1.089 (1.089, 1.090) < 0.00001	0.119	1.0	0.930 (0.928, 0.931) < 0.00001	0.537
Non-Hispanic Black	1.0	0.875 (0.874, 0.877) < 0.00001	0.211	1.0	0.925 (0.923, 0.926) < 0.00001	0.291	1.0	0.729 (0.726, 0.731) < 0.00001	0.076
Other Race	1.0	0.955 (0.953, 0.957) < 0.00001	0.789	1.0	0.992 (0.990, 0.994) < 0.00001	0.805	1.0	0.870 (0.862, 0.878) < 0.00001	0.700

Crude model: no adjustment;

Model I: adjusted 1: age, races; 2: gender, races; 3: gender, age;

Model II: further adjusted education, average alcohol intake, average cigarettes, marital status

There is also an association between sleep duration and the incidence of wrist fractures among different sex groups (P for trend < 0.001). When divided into quartile based on age, there was no significant difference in the incidence of wrist fractures among different age groups based on sleep duration (P for trend all > 0.05). According to different ethnicity of patients, there was a difference in the incidence of wrist fractures based on sleep duration (Table 4).

## Discussion

In 2000, the World Health Organization reported that the prevalence of wrist fractures in people aged 50 and over was 18.5% [9]. This study found that shorter sleep duration leads to an increased incidence of wrist fractures in middle-aged and elderly people over 50 years old. Possible reasons for this include: (a) Short sleep duration leads to autonomic sympathetic dysfunction, which leads to the destruction of bone turnover markers and the loss of cortical bone thickness [8, 11]; (b) Sleep can also have a significant impact on bone health through hormonal regulation, with the main hormones including lepton and growth hormone [8, 12]. The relationship between sleep duration and bone health has also been observed in animals. Sleep restriction and long-term sleep deprivation lead to decreased markers of bone formation and bone resorption in rats [13, 14]; (c) Short sleep duration may cause patients to become distracted and unable to respond promptly during trauma, leading to the occurrence of wrist fractures. In order to alleviate the adverse effects of short sleep duration on bone health, the National Sleep Foundation recommends sleeping for more than 7 h per day [10].

In addition, our study found that the association between sleep duration and wrist fractures varies greatly among ethnic groups, but the difference is not significant, which is different from some previous studies [15]. The advantages of our study include the use of large sample sizes from multiple years across the country, as well as the use of multivariate adjusted models to control for relevant confounding factors. In subgroup analyses, groups characterized by age, gender, and ethnicity were evaluated carefully. Our data can help establish an appropriate strategy to improve sleep duration and prevent wrist fractures. It is recommended that adequate sleep education be provided to a wide range of patients, and early identification of these potentially frail groups will help us reduce the incidence of wrist fractures in the population based on healthy sleep management in the future, and improve the quality of life of middle-aged and elderly patients. Provide evidence for clinical patients to manage healthy sleep.

Despite this, there are still some limitations to this study. Our study lacked an assessment of sleep quality, and there is currently no precise method for evaluating sleep quality. The next step is to appropriately supplement the questionnaire to further enrich the evidence for healthy sleep.

## Conclusion

A negative association between sleep duration and wrist fractures has been observed in the adult population in the United States. Our findings provide a better understanding of the relationship between sleep duration and wrist fractures. Further research can be conducted based on existing data to elucidate the mechanisms behind this relationship.

## Data Availability

The datasets generated and analysed during the current study are available in the NHANES data (www.cdc.gov/nchs/nhanes), or required from the corresponding author.
